# Ovarian tissue transplantation ameliorates osteoporosis and dyslipidaemia in ovariectomised mice

**DOI:** 10.1186/s13048-022-01083-0

**Published:** 2022-12-28

**Authors:** Encheng Zhou, Du Xiang, Bin Yu, Hanlin Yao, Chao Sun, Yanfeng Wang

**Affiliations:** Zhongnan Hospital of Wuhan University, Institute of Hepatobiliary Diseases of Wuhan University, Transplant Center of Wuhan University, National Quality Control Center for Donated Organ Procurement, Hubei Key Laboratory of Medical Technology on Transplantation, Hubei Clinical Research Center for Natural Polymer Biological Liver, Hubei Engineering Center of Natural Polymer-based Medical Materials, 430071 Wuhan, China

**Keywords:** Ovarian tissue transplantation, Hormone replacement therapy, Osteoporosis, Dyslipidaemia, Endocrine function

## Abstract

**Background:**

Ovarian insufficiency frequently renders postmenopausal women susceptible to osteoporosis and dyslipidaemia. Postmenopausal transplant women are at a higher risk developing osteoporosis and dyslipidaemia due to the concomitant application of glucocorticoids and immunosuppressants after solid organ transplantation. Thus, this study aimed to explore the feasibility of ovarian tissue transplantation (OTT) as an alternative to Hormone replacement therapy (HRT) for postmenopausal women with solid organ transplant needs.

**Results:**

Sixty mice were randomly divided into four groups: sham operation, ovariectomised (OVX group), ovariectomy plus oestrogen (E_2_ group), and ovariectomy plus OTT (OTT group). The inhibin levels in the OTT group were increased and the follicle stimulating hormone and luteinizing hormone were suppressed to normal levels, which could not be achieved in the E_2_ group. The femoral bone mineral density in the OTT group was significantly increased than the E_2_ group (*P* < 0.05), and the probability of fracture was reduced by 1.4–2.6 times. Additionally, the high-density lipoprotein cholesterol levels were higher in the OTT group than in the E_2_ group and the triglyceride levels were lower in the OTT group than in the E_2_ group (*P* < 0.05).

**Conclusion:**

OTT not only achieves certain endocrine effects by participating in the regulation of the hypothalamic-pituitary-ovarian feedback control loop, but also ameliorates osteoporosis and dyslipidaemia, which may be an alternative to traditional HRT for postmenopausal women with solid organ transplant needs.

## Background

As life expectancy is increasing, naturally menopausal women will be in menopause for about one third of their lifetime, with an increased risk of various complications related to oestrogen (E_2_) deficiency and elevated gonadotropins, such as osteoporosis, atherosclerosis, dyslipidaemia, obesity, and genital atrophy [1–3]. Osteoporosis affects approximately 50% of postmenopausal women worldwide, and the prevalence of fractures among people with osteoporosis is as high as 40% [4, 5]. Other studies have shown that postmenopausal women develop various dyslipidaemia disorders due to hormonal changes, which significantly elevates the risk of atherosclerosis and stroke [6, 7]. Thus, improving the management of menopausal syndromes in postmenopausal women is undoubtedly becoming a challenging clinical issue worldwide.

Various efforts have been made to replace degenerative ovarian function, including hormone replacement therapy (HRT). HRT remains the most widely used treatment for postmenopausal women [8, 9], and extensive work has been done to assess the balance of risk/benefit of HRT showing that with a proper patient stratification of risk, the benefits overweigh the risks [10, 11]. However, along with the number of transplants performed per year and the life expectancy of transplant recipients is steadily increasing. Postmenopausal transplant women, as a specific population of postmenopausal women, are at a higher risk developing osteoporosis and dyslipidaemia due to the concomitant application of glucocorticoids and immunosuppressants after solid organ transplantation [12–14]. Furthermore, careful clinical monitoring together with regular surveillance of biochemical parameters are recommended in transplanted HRT users as HRT may lead to deterioration of transplanted organ function [15, 16]. Therefore, besides traditional HRT, there is a need for an alternative that not only maintains the beneficial effects but also improves safety for this specific population. It is well established that ovarian tissue transplantation (OTT) can offer effective strategies for degenerative ovarian function, hormone levels in plasma can be detected following implantation of ovarian tissue, and hormone secretion lasts for more than five years [17–19]. Moreover, for postmenopausal women with solid organ transplant needs, it is feasible to transplant a few slices of allogeneic ovarian tissue at the same time as the solid organ transplantation without additional immunosuppressants, which may provide a viable hormone replacement option for postmenopausal transplant women.

However, there is little research on OTT as a potential hormone replacement approach [20]. This study used an ovariectomized (OVX) mouse model to simulate the endocrine characteristics of postmenopausal women that were later transplanted with ovarian tissue. Femoral micro-computed tomography (Micro-CT), histology, serum endocrine and lipid hormone levels, and uterine and vaginal epithelial cell morphology, were used to evaluate the therapeutic effects of OTT on osteoporosis and dyslipidaemia, providing an experimental basis for OTT as a viable hormone replacement option for postmenopausal transplant recipients in the future.

## Materials and methods

### Animals and groups

Female BALB/c mice obtained from Beijing Wei Tong Li Hua Bio-Technology Co., Ltd. (Beijing, China) were used in the present study. Ten-to twelve-day-old immature female mice were used as donors for ovarian tissue, while six-to eight-week-old mature female mice served as allogeneic transplant recipients. Recipients underwent bilateral OVX, after the baseline of circulating E_2_ was confirmed, OVX mice were classified into three groups (OVX mice, OVX mice received E_2_, OVX mice implanted with ovarian tissue), The HRT treatment was injected subcutaneous given at a dosage of 25 µg/kg/d of E_2_. This dose was selected based on previous studies [21–23]. The animals were randomly divided into four groups (*n* = 15/group). OVX mice were used as negative controls, whereas sham-operated mice served as positive controls.

### Construction of the OTT mouse model

All operations were performed under pentobarbital sodium anaesthesia (60 mg/kg, intravenously). Ketamine (10 mg/kg, intravenous) was administered postoperatively to control postoperative pain. Two weeks post-OVX, the ovary on one side of the donor was removed and cut equally into two pieces, one of which was placed within the anterior abdominal wall of the recipient. Mice were administered a subcutaneous injection of 100 mL/kg of warm saline after surgery to assist in maintaining hydration. Throughout the procedure, the mice were placed on a hot surgical pad (37 °C) until they were fully awake. The transplanted mice were sampled for further testing after six weeks of the surgery.

### Measurement of plasma E_2_, luteinizing hormone (LH), follicle stimulating hormone (FSH), and inhibin

To assess the recovery of endocrine function in OVX mice transplanted with ovarian tissue, serum endocrine hormone levels were measured in all four experimental groups of mice every two weeks, including E_2_, follicle stimulating hormone (FSH), luteinizing hormone (LH), and inhibin. Hormone levels in plasma samples were evaluated using ELISA kits in accordance with the manufacturer’s instructions. The E_2_ (2CEA461Ge), FSH (CEA830Mu), LH (CEA441Mu) and inhibin (SEA760Mu) were all detected using competitive ELISA kits purchased from Cloud-Clone Corp (Wuhan, China). None of the competitive ELISA kits reported cross-reactivity.

### Cytology of vaginal exfoliation in mice

Vaginal secretions were collected two weeks after transplantation (between 8 and 11 am) to observe the motility cycle of the mice. Vaginal secretions were obtained using a plastic pipette containing 100 µL of saline (NaCl, 0.9%) and the tip was inserted shallowly into the vagina of the mice. The vaginal fluid was then placed inside a pipette on a glass slide. One drop was collected from each mouse sample using a clean pipette and, a different glass slide for each mouse. The stained sections were observed under a light microscope at 10× and 40×. Three types of cells were identified under a microscope: irregular and without nuclear cornified cells, round and nucleated epithelial cells, and small round leukocytes. The proportions among them were used for the determine the oestrous cycle phase.

### Femur bone micro-CT scans

Micro-CT scans were used to measure femoral bone mineral density (BMD) and the microstructure of bone trabeculae at six-weeks after transplantation. Femur bones were removed from each mouse after the research was completed, and their qualities were analysed using a SKYSCAN 1276 CMOS X-ray Micro-CT (Bruker, Belgium). After washing the femur with saline, the distal femoral region was scanned using Micro CT at a thickness of 18 μm. Three-dimensional images of the skeleton were established, and the following parameters were analysed: BMD [g/cm^^3^], fractional trabecular bone volume (BV/TV [%]), trabecular thickness (Tb. Th [µm]), trabecular number (Tb. N [1/mm]), trabecular separation (Tb. Sp [µm]), and structure model index (SMI).

### Femur bone histology and immunohistochemistry

Evaluation of restoration of bone metabolic balance after OTT. After micro-CT scanning, the femurs were decalcified in 10% EDTA, dehydrated, and embedded in paraffin. Paraffin was sectioned to a thickness of 4 mm. After staining with H&E, the sections were observed under a light microscope at 10× and 40× magnifications. The sections were also subjected to tartrate-resistant acid phosphatase (TRAP) and immunohistochemical (IHC) staining using TRAP/ALP Stain Kit (294-67001, Wako Pure Chemical Industries, Osaka, Japan). Primary antibodies against MMP9 (10375-2-AP, Proteintech) and NFATc1 (66931-1-Ig, Proteintech) were used for immunohistochemistry. TRAP-stained cells and, MMP9 and NFATc1 positive cells were analysed using Image J in the sections from each mouse.

### Body weight, abdominal fat weight and plasma levels of lipid

Mice were weighed every two weeks, and blood samples were collected for 8 weeks to measure hormonal plasma levels, including total cholesterol (T-CHO), triglyceride (TG), high-density lipoprotein cholesterol (HDL-C), and low-density lipoprotein cholesterol (LDL-C). Abdominal fat (transverse septum as the upper boundary and pelvic floor fascia as the lower boundary) was explanted from each mouse after the research was completed. The levels of T-CHO, TG, HDL-C, and LDL-C were evaluated in accordance with the manufacturer’s instructions using ELISA kits (Jian Cheng Bio-technology and Science Inc. Nanjing, China).

### Statistical analysis

Data were analysed and processed using GraphPad Prism 8.0 (GraphPad Software, Inc.) and SPSS version 12.0 software (SPSS, Inc.). Statistical analyses were performed using one-way analysis of variance (ANOVA) with Tukey’s post-hoc test. Continuous variables are represented as mean ± standard deviation (SD). Statistical significance was set at *P* < 0.05.

## Results

### OTT restored endocrine function byparticipating in the hypothalamic-pituitary-ovarian (HPO) feedback control loop

**Fig. 1:**
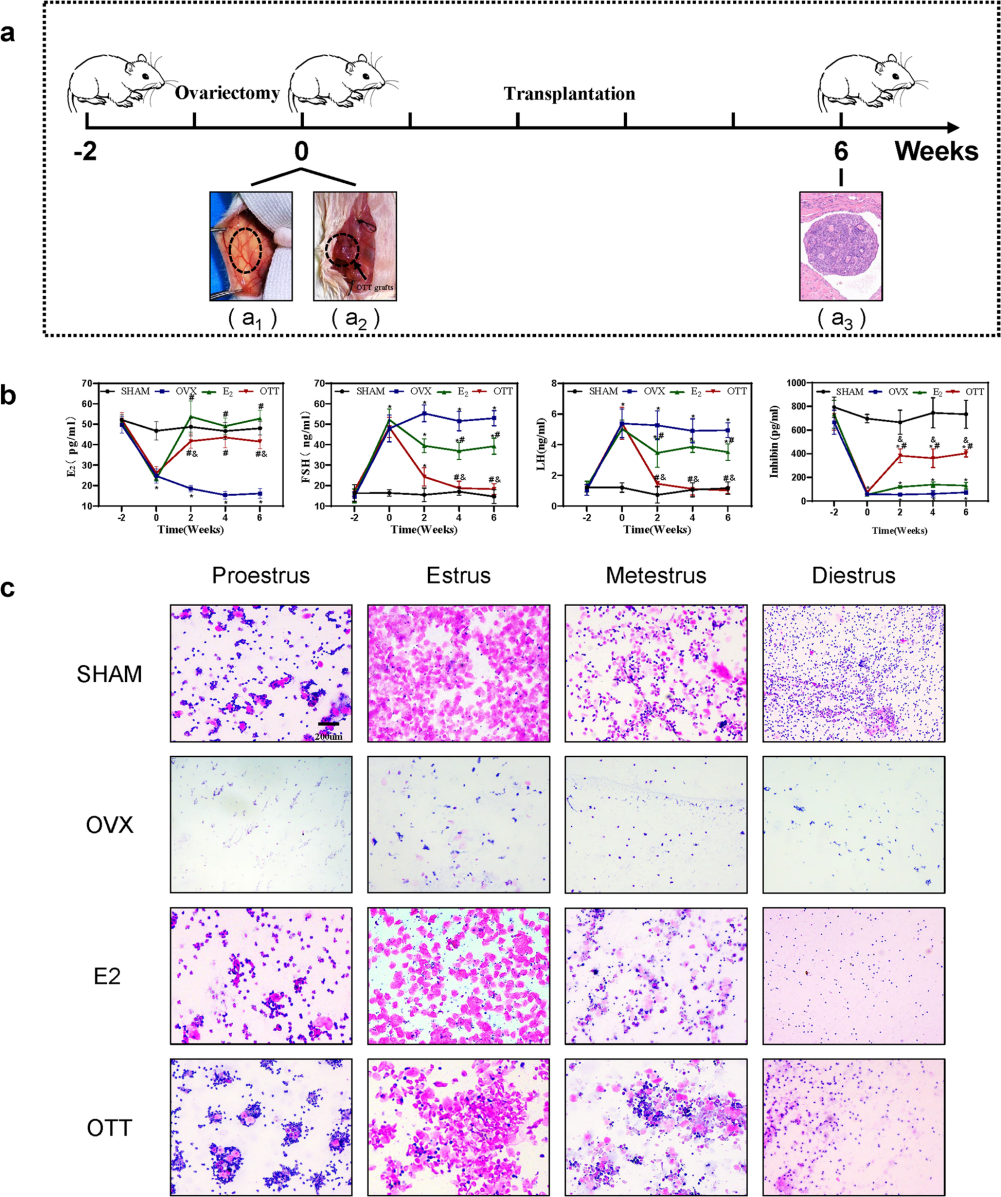
Experimental process and the endocrine function of the OTT.** a** Schematic illustration of the animal experiment. (a_1_) Transplantation of ovarian tissue into the peritoneum of ovariectomised mice. (a_2_) Ovarian tissue in the peritoneal pouch after transplantation, implanted ovarian tissue is shown in the red box. (a_3_) Images of ovarian tissue haematoxylin and eosin staining. **b** Changes in the levels of serum E_2_, FSH, LH and inhibin in the four groups. **c** Representative images of the four groups of vaginal cytology are as follows: SHAM, OVX, E_2_ and OTT. * indicates significance *P* < 0.05 compared to normal mice; # indicates significance *P* < 0.05 compared to OVX mice; & indicates significance *P* < 0.05 compared to E_2_ mice as determined by a two-way ANOVA followed by Tukey’s post hoc analysis

The potential strength of OTT is its capacity to engage in the HPO feedback control loop to regulate plasma endocrine hormone concentrations. As shown in Fig. 1b, plasma E_2_ levels in mice receiving OTT and E_2_were dramatically higher than those in OVX mice, while E_2_ levels were slightly higher in HRT mice and slightly lower in OTT mice than in sham mice. We also assessed plasma concentrations of inhibin, FSH and LH. Both FSH and LH levels in the plasma of HRT mice increased after treatment, which is known to be due to the absence of the HPO feedback control loop. It is apparent from our results that HRT did not fully inhibit the increased FSH and LH to normal levels. In contrast, the levels of FSH and LH in OTT mice were completely suppressed (*P* < 0.05), and their levels were equivalent to those in sham-operated mice. Furthermore, the levels of inhibin produced by OTT ranged between the levels of OVX and sham-operated mice, whereas HRT was the same as OVX. The Fig. 1c shows the vaginal smears of each group of female mice, and we determined the stage of the oestrous cycle based on the proportion of each cell [24]. As shown in Fig. 1c, both OTT and HRT treatments restored the oestrous cycle in mice, which disappeared in normal mice two weeks post-OVX.

### OTT prevented OVX-induced bone mass loss

**Fig. 2:**
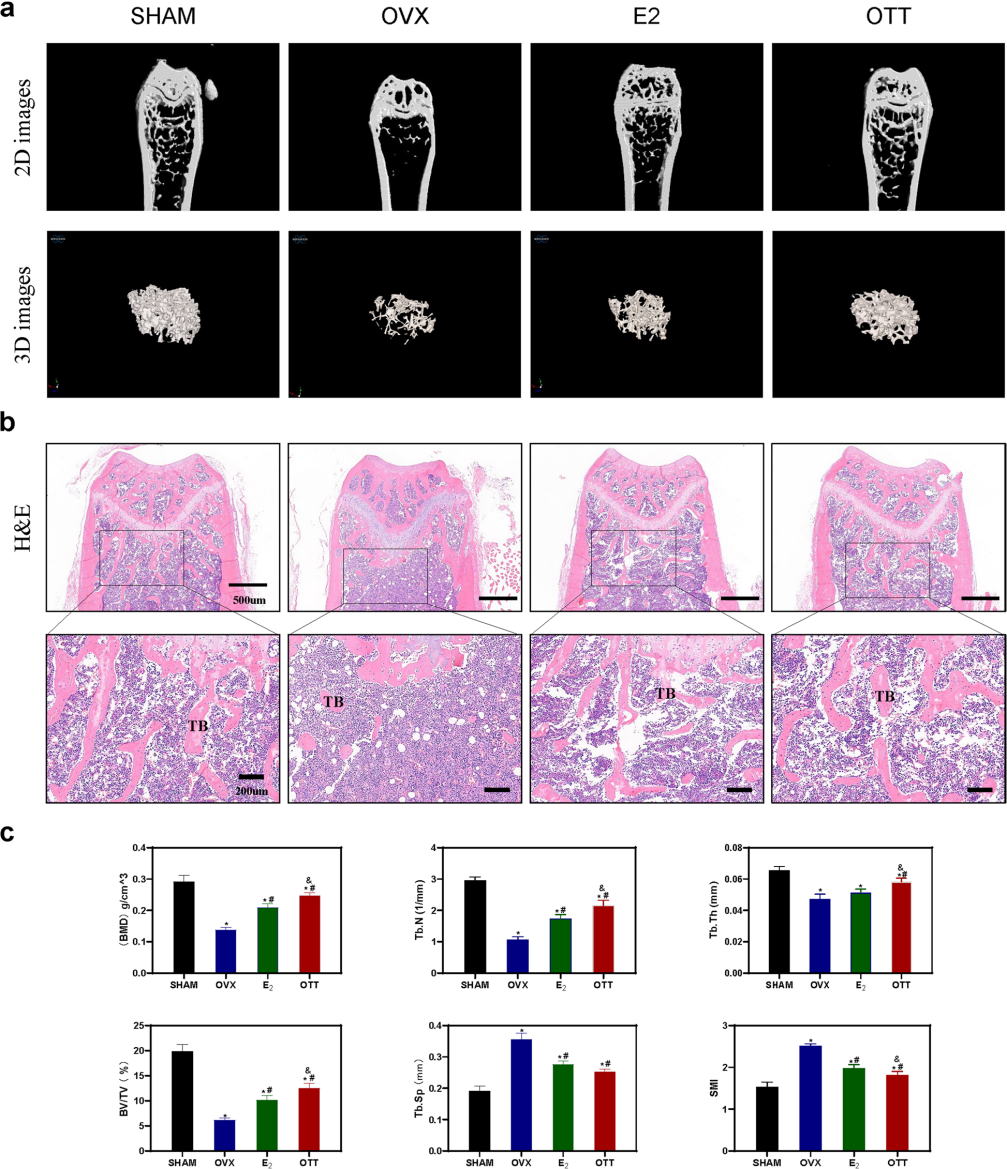
Femur bone Micro-CT scans and histology.** a** Micro-CT analysis of distal femur in each group. **b** Haematoxylin and eosin staining in each group. **c** Quantitative analysis of bone mineral density (BMD), trabecular number (Tb.N), trabecular thickness (Tb.Th), bone volume/tissue volume(BV/TV), structure model index (SMI) and trabecular separation (Tb.Sp). * indicates significance *P* < 0.05 compared to normal mice; # indicates significance *P* < 0.05 compared to OVX mice; & indicates significance *P* < 0.05 compared to E_2_ mice as determined by one-way ANOVA followed by Tukey’s post hoc analysis

The micro-CT scans and H&E staining shown in Fig. 2 indicate differences in the BMD and trabecular bone microarchitecture between the different groups. Notably, bone from mice transplanted with ovarian tissue showed a morphology and histology similar to that of normal bone, with a greater increase in BMD and better trabecular structural arrangement and continuity. Mice that received E_2_ showed less bone mass loss than OVX mice, but with smaller BV/TV, thinner Tb.Th and lower Tb.N compared to OTT group (*P* < 0.05). The OTT group achieved BMD levels that were higher than the E_2_ group (*P* < 0.05), which is specifically compelling given that the OTT had lower plasma E2 levels in circulation than HRT regimens. In short, although Tb.Sp levels were similar in OTT and HRT, BMD, BV/TV and Tb.N was superior in the OTT group. Generally, the morphological and histological results presented in Fig. 2 suggest that better skeletal outcomes were achieved with OTT than HRT treatment.

### OTT inhibited osteoclast-mediated bone resorption

**Fig. 3:**
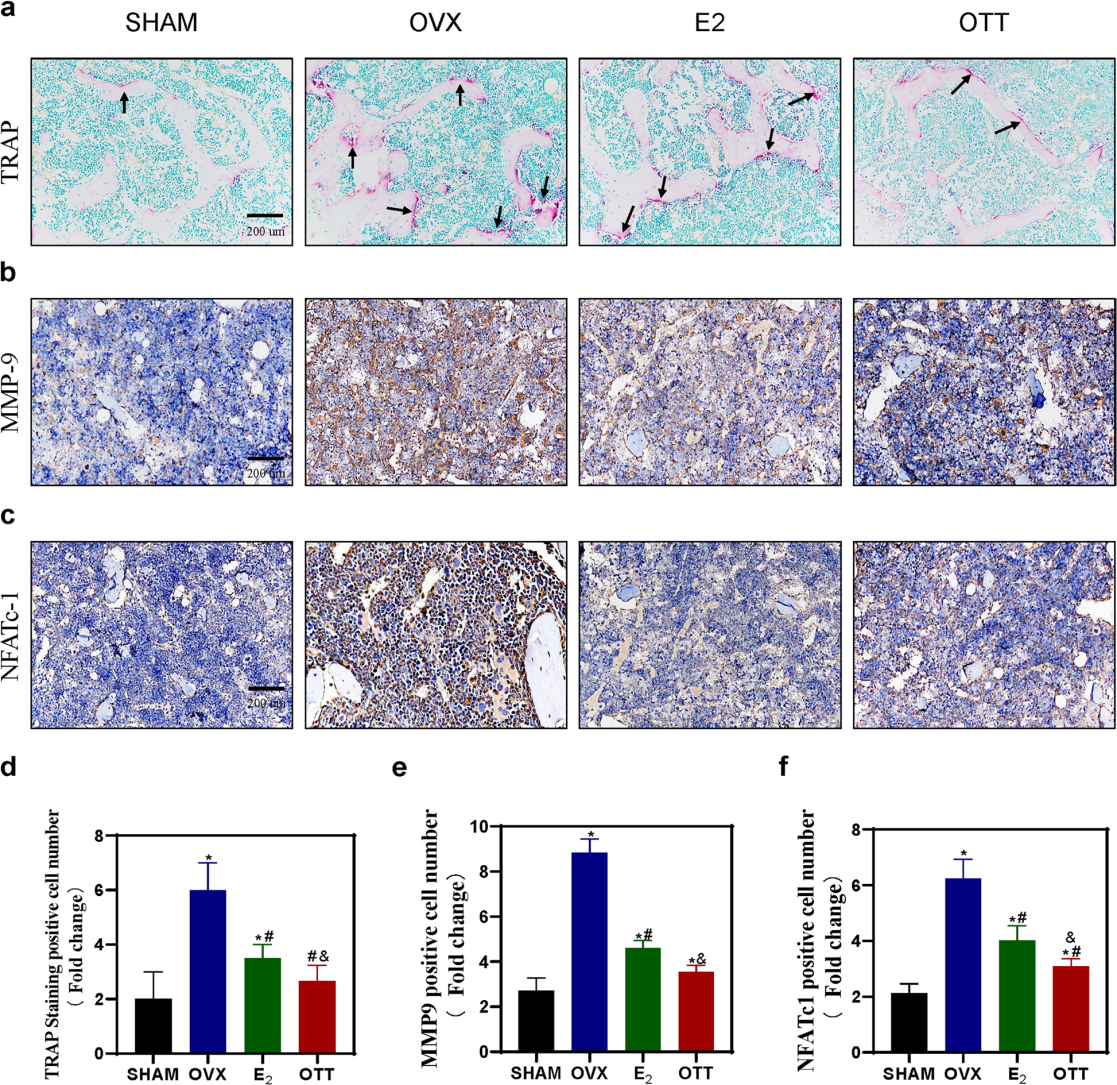
TRAP staining and IHC. **a** TRAP staining of femur bone sections in each group. **b-c** Representative images show the IHC staining of the femur bone sections with antibodies against bone resorption markers, MMP9 and NFATc1. **d** Quantitative analysis of TRAP, MMP9 and NFATc1 positive cells in the femur bone sections; * indicates significance *P* < 0.05 compared to normal mice; # indicates significance *P* < 0.05 compared to OVX mice; & indicates significance *P* < 0.05 compared to E_2_ mice as determined by one-way ANOVA followed by Tukey’s post hoc analysis

We evaluated the influence of OTT on metabolic biomarkers of the bone in Fig. 3. TRAP staining of bone tissue showed a significant reduction in osteoclasts in both OTT and E_2_ group mice compared to the OVX group; however, animals that received HRT showed higher levels of positive cells than sham and OTT group (*P* < 0.05). Particularly, the OTT group was similar to the sham group in terms of osteoclast number and osteoclast area on the bone surface **(**Fig. 3a).

MMP9 and NFATc1 have been identified as major regulators of osteoclastogenesis in vivo [25]. IHC of bone resorption markers indicated a significant increase in the number of MMP9 and NFATc1 positive cells in the OVX group compared with the other groups, but a decrease in the OTT and E_2_ groups compared with the OVX group **(**Fig. 3b). Moreover, the number and surface area of positive cells was slightly less in the OTT than E_2_ group that is closer to the sham group (*P* < 0.05).

Overall, the results presented in Fig. 3 suggest that OTT is superior to HRT in terms of bone metabolism biomarkers by inhibiting the proliferation of TRAP positive osteoclasts and suppressing the bone resorption activity of osteoclasts.

### OTT decreased body fat and plasma lipid levels

**Fig. 4:**
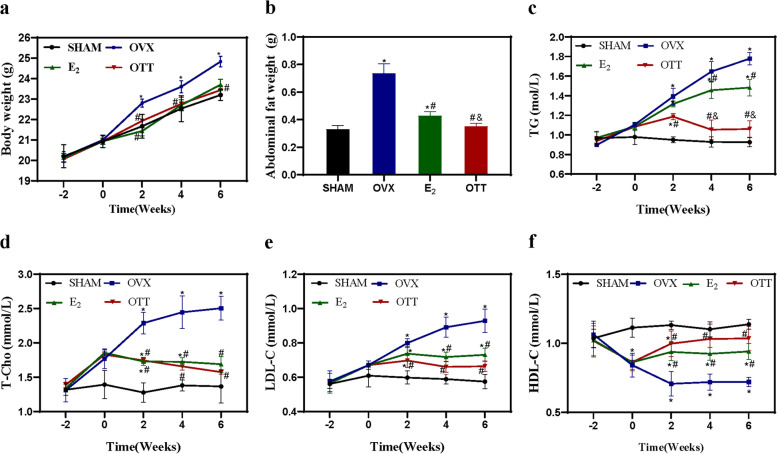
Body weight, abdominal fat weight and plasma levels of lipid.** a** Changes in the levels body weight in each group. **b** The abdominal fat weight before sacrifice (6 weeks after transplantation) in each group. **c-f** Changes in the levels of serum TG, T-CHO, LDL-C and HDL-C in the four groups. * indicates significance *P* < 0.05 compared to normal mice; # indicates significance *P* < 0.05 compared to OVX mice; & indicates significance *P* < 0.05 compared to E_2_ mice as determined by one-way ANOVA (a, c-f) or two-way ANOVA (b)

We evaluated the effects of HRT and OTT on body weight using weekly monitoring. As revealed in Fig. 4, OVX mice receiving E_2_ and OVX mice treated with the OTT had body weight and abdominal fat weight comparable to the levels in sham group. The Fig. 4c-f indicates the different serum lipid levels between the various treatments. Both OTT and E_2_ group had four lipid profiles that were improved compared with OVX mice. Interestingly, the levels of TG observed with HRT therapy were higher than those observed with OTT therapy (*P* < 0.05), which may explain why HRT increased the risk of cardiovascular diseases such as atherosclerosis. Furthermore, the levels of HDL-C in animals receiving the OTT were higher than that observed with HRT, and no statistical difference in HDL-C between OTT group and sham group.

## Discussion

Osteoporosis and dyslipidemia are plaguing an increasing number of postmenopausal women. HRT is currently the main treatment modality for postmenopausal women to relieve the symptoms. However, postmenopausal transplant women, as a specific population of postmenopausal women, are at a higher risk developing osteoporosis and dyslipidaemia due to the long-term application of glucocorticoids and immunosuppressants after solid organ transplantation. Furthermore, deterioration of transplanted organ function has been reported among transplanted HRT users, this side effect of HRT may be associated with impaired metabolism of drugs [12]. Therefore, the ovarian tissue-based hormone replacement system illustrated in this study may provide an attractive alternative and is consistent with the recommended hormone dose in replacement therapy suggested by the current US and European guidelines [26, 27]. In a comprehensive development, the technique discussed throughout this article caters primarily to postmenopausal women who simultaneously require solid organ transplantation, like kidney, heart and liver.

Ovary transplantation can be divided into orthotopic and heterotopic transplantation, it was proven that the latter has more outstanding advantages if not for fertility restoration: it mainly overcomes the complexity of vascular anastomotic transplantation, because the graft site is superficial and easy to observe and handle the graft [28, 29]. The peritoneum has these specifications and is considered a common implantation site for subcutaneous transplantation [30–32]. As shown in our study, the heterotopic site may be an accessible and convenient location for transplantation, such as the peritoneum, which makes the surgery less invasive, possibly achieved with local anaesthesia, and feasible even in the presence of severe pelvic adhesions. Therefore, heterotopic OTT may be more feasible for clinical application in transplant recipients.

Although the plasma E_2_ levels reached by OTT were slightly lower than those of normal mice were, they effectively inhibited FSH and LH levels in OVX mice. The evaluated inhibin concentrations in transplanted mice also confirmed previous reports of inhibin secretion [33]. It is well known that E_2_ and inhibin have feedback regulatory effects on gonadotropin-releasing hormone secreted by the hypothalamus, an interpretation of our results is that, in contrast to HRT, OTT restored the HPO axis in OVX mice. These findings highlight the opinion that the levels of ovarian endocrine hormones produced by ovarian tissue are of physiological relevance, and effective terminal organ effects are achieved at lower and more secure plasma levels. Other ovarian hormones secreted by the OTT, such as FSH and inhibin, have broader applications that are not available with HRT. For instance, osteoporosis related to postmenopausal symptoms has been reported to be not only due to a decrease in E_2_ but may also be associated with a decrease in inhibin and increased FSH levels [34–36]. Disruption of the HPO feedback control loop leads to elevated FSH levels, and consequently, increased osteoclastogenesis and bone loss [37, 38]. The secretion of inhibin could also explain our results, with FSH maintained at low levels despite a slightly lower E_2_ secretion. Furthermore, FSH is also an important factor in the regulation of lipid synthesis, elevated FSH levels could directly lead to adiposity and hypercholesterolaemia, which is an important reason why postmenopausal women show a high risk of developing dyslipidaemia [39, 40]. In summary, all these instances emphasise the importance of other hormones, as well as factors produced by the ovary, which may only be provided through OTT. However, in the present study, inhibin in the OTT group was restored to approximately half of the normal level, which may reflect that only part of the follicle survived after OTT, as inhibin is also used as an important indicator of ovarian reserve [41]. Moreover, considering that elevated levels of FSH after ovariectomy hindered follicle development, these may account for the lower inhibin in this study.

To the best of our knowledge, the effects of OTT on postmenopausal syndromes such as osteoporosis and dyslipidaemia, have not been adequately evaluated. Therefore, according to the WHO classification of osteoporosis [42], our study showed that the OTT group had a T-score of -2.2, which is classified as osteoporosis, and the E_2_ group had a T-score of -4.1, which is classified as severe osteoporosis, possibly indicating a 1.4–2.6 factor reduction in the probability of fracture in the OTT group compared to the E_2_ group [40]. Besides hormones that contribute to the amelioration of osteoporosis, many studies have identified a link between dyslipidaemia and low BMD in postmenopausal women [43–46]. We observed that OTT may slight better than HRT in improving some of the metabolic effects such as hypertriglyceridaemia. Moreover, a recent study suggested that cholesterol might directly act through osteoclasts to induce bone loss in postmenopausal women [47]. In our study, the OTT group had lower TG levels and higher BMD than the E_2_ group. Therefore, OTT could better restore both unbalanced plasma lipid levels and bone loss after menopause. Taken together, OTT may perform better than HRT in terms of ameliorating osteoporosis and dyslipidaemia, indicating that OTT may provide a viable option for postmenopausal women with solid organ transplant needs (e.g., kidney, heart and liver).

However, this study has certain limitations. First, although the 6-week duration OVX model of osteoporosis is consistent with other investigations [48–50], longer-term studies, such as a 2–3 months mouse model, will be considered in the future. In addition, considering that ovariectomised animal model does not undergo natural aging, natural menopausal animal model will be used in the future. Furthermore, solid organ transplantation in combination with OTT will be of interest in the future.

## Conclusion

In summary, the use of OTT as a novel approach to hormone replacement is interesting for postmenopausal women with solid organ transplant needs, not only to increase the range of options for women requiring HRT, but also to highlight the potential use of OTT in the treatment and prevention of diseases related to postmenopausal symptoms. Furthermore, the results generated in this study need to be further validated in clinical trials.

## Data Availability

The datasets used and/or analyzed during the current study are available from the corresponding author upon reasonable request.

## Abbreviations

OTT: Ovarian tissue transplantation
HRT: Hormone replacement therapy
HPO: Hypothalamic–pituitary–ovarian
OVX: Ovariectomised
E_2_: Oestrogen
FSH: Follicle stimulating hormone
LH: Luteinizing hormone
TG: Triglyceride
Micro-CT: Micro-computed tomography
T-CHO: Total cholesterol
HDL-C: High-density lipoprotein cholesterol
LDL-C: Low-density lipoprotein cholesterol
BMD: Bone mineral density
BV/TV: Fractional trabecular bone volume
Tb. Th: Trabecular thickness
Tb. N: Trabecular number
Tb. Sp: Trabecular separation
SMI: Structure model index
TRAP: Tartrate-resistant acid phosphatase
ANOVA: Analysis of variance
IHC: Immunohistochemical
H&E: Haematoxylin and eosin
